# Evaluation of the correlation between oral infections and systemic complications in kidney transplant patients: a retrospective pilot study

**DOI:** 10.1186/s12903-022-02590-8

**Published:** 2022-11-24

**Authors:** Renato Pol, Davide Camisassa, Marta Bezzi, Luca Savoldi, Federica Punzi, Massimo Carossa, Tiziana Ruggiero

**Affiliations:** 1 Department of Surgical Sciences, C.I.R. Dental School, University of Turin, 10126 Turin, Italy; 2 Politecnico di Torino, Corso Duca degli Abruzzi 24, 10129 Turin, Italy

**Keywords:** Dental clearance, Kidney transplantation, Odontogenic foci, Oral bacteria, Oral health, Systemic complications

## Abstract

**Background:**

Data regarding the efficacy of the dental clearance required prior to kidney transplantation (KT) for preventing post-transplant complications is controversial. The aim of this retrospective study was to investigate a possible correlation between any untreated oral infectious foci and the onset of systemic complications in KT patients.

**Methods:**

Patients scheduled for regular check-ups during the post-transplant period were visited at the C.I.R. Dental School in Turin, Italy. Patients were asked to bring orthopantomography (OPT) acquired prior to transplantation to compare the possible presence of untreated infectious foci at the time of transplantation with the time of their post-transplant visit. Patients were then divided, according to the evaluation of the OPT obtained prior to the transplantation, into two groups according to their dental status prior to the transplant. “Group Infected” was comprised of patients with no dental clearance, and “Group Clear” included patients with dental clearance. The medical records were then retrospectively reviewed for the evaluation of any systemic complications that occurred after transplantation. The following medical complications were considered: fever, pneumonia, urinary tract infections, systemic infections, kidney rejection, and death. Complications were divided in two groups: early complications, which occurred within 100 days of transplantation, and late complications, which occurred more than 100 days after transplantation.

**Results:**

A total of 77 patients were enrolled in the study. Group Infected was composed of 19 subjects (25%), while Group Clear was composed of 58 patients (75%). In Group Infected, 13 (68%) patients developed complications within 100 days of transplantation, and 11 (58%) did so after 100 days. In Group Clear, 31 (53%) patients had complications within 100 days of the transplant, and 23 (40%) did after 100 days. Patients in Group Infected had a statistically significant increase in episodes of fever (*p* = 0.03), compared to Group Clear, with a higher relative risk (RR) of 3.66 in the first 100 days after transplantation.

**Conclusion:**

Within the limitations of the present retrospective pilot study, and based on the results, a correlation between the absence of dental clearance prior to KT and a higher RR of developing a fever within the first 100 days post transplantation was highlighted. The present results encourage doctors to continue research on the topic, which remains controversial. Further prospective studies are required to confirm the results of the present study.

## Background

Kidney transplantation (KT) is considered the treatment of choice for patients with chronic, end-stage kidney failure [1, 2]. The procedure replaces organ function and provides patients with a better quality of life than patients on dialysis, resulting in an increase in physical independence and, particularly, a reduction in mortality rates [3]. The excellent results obtained in KT patients are largely attributed to advances in immunosuppression. The primary purpose of immunosuppression drugs is to prevent organ rejection and safely preserve allograft function [4]. This category of drugs acts by inhibiting the immune system's response to non self antigens, so that the immune system itself does not affect the newly transplanted foreign organ. However, this also implies a lower capacity of the organism to fight infectious agents [4, 5]. In fact, infections are considered one of the most common causes of graft failure and death after transplant [6–9].

The oral microbiome can be classified as a “core microbiome,” which is similar for all humans, and a “variable microbiome,” which is closely related to the lifestyle and the phenotypic and genotypic determinants of the individual [10, 11]. It has been recently estimated that the core microbiome is composed of more than 700 species, making it the second-most complex microbiome of the human body [12]. Nevertheless, it represents a passageway between the external environment and the organism through the respiratory and digestive tracts. Consequently, research has started to point out a possible influence of the oral microbiome on systemic diseases [13]. Dominy et al. [14] provided evidence in the brains of patients with Alzheimer’s disease of the presence of bacteria commonly implicated in gum disease. Bergandi et al. [15] demonstrated a correlation between apical periodontitis and increased endothelial dysfunction markers. Further, two recent systematic reviews [16, 17] analyzed the interrelation between the oral environment, psychological aspects, and systemic conditions. In both reviews, it was highlighted how the three parameters evaluated were often interrelated and capable of influencing the patient’s quality of life.

In regard to kidney disease, the first report that hypothesized a possible correlation between oral infections and complications in KT patients was published in 1982 [18]. Since then, research has started to investigate the possible role of dental infections and complications in KT patients, and many transplant hospitals have started to require a so-called “dental clearance” prior to surgery. Dental clearance is commonly defined as the complete absence of any active dental pathology or source of infection in the oral cavity. In a recent systematic review on the topic, Schmalz et al. [19] suggested how a multidisciplinary approach, which includes dentists, should be always adopted both prior to and after kidney replacement. Sarmento et al. [20] found a correlation in the short term between a poor dental environment and an increase in hospital length of stay after KT. Zwiech et al. [21] highlighted an association between dental infections and an increased risk of systemic complications during the first year after KT. However, other authors have questioned the true efficacy of dental clearance for preventing KT complications, pointing out that the scientific evidence is weak [22, 23]. Consequently, the topic remains controversial.

With the above in mind, the first aim of this retrospective pilot study was to investigate a possible correlation between any untreated oral infectious foci and systemic complications in KT patients, reporting on data acquired over five years of study. The second objective was to evaluate the presence or absence of typical oral pathogenic bacteria in transplanted patients who experienced systemic infections.

The null hypotheses were: (1) there is no correlation between untreated oral infections and post-surgical complications in patients who underwent KT and (2) oral cavity bacteria are not systemically present in patients who experienced systemic infections after KT.

## Methods

### Study characteristics, participants, and design

Between June 2016 and June 2021, patients who were scheduled for regular follow-ups during the post-transplant period were seen at the C.I.R. Dental School in Turin, Italy. The “dentistry supportive care” protocol after KT includes: (1) regular oral visits, including dental, periodontal, and oral mucosal condition assessment via physical examination within three months after the KT and one year after; (2) professional oral hygiene care when needed; and (3) radiographic analysis by orthopantomography (OPT), required on the day of the visit. Additionally, for the retrospective purpose of the study, patients were asked to bring OPT acquired prior to transplantation to compare the possible presence of untreated infectious foci at the time of transplantation with the time of the post-transplant visit. OPT is routinely required two months prior to transplant surgeries, in accordance with the pre-transplant protocol of the hospital Città della Salute Molinette, in Turin. Additionally, dental records were investigated to determine if any infected pockets (probing depth > 3 mm) were noticed at the moment of the pre-KT dental visit. The nephrological medical records for all KT patients were retrospectively reviewed for the evaluation of systemic complications that occurred after transplantation. Complications were divided in two groups: early complications, those occurring within 100 days after transplantation, and late complications, occurring more than 100 days after transplantation. The following medical complications were considered: fever, pneumonia, urinary tract infections, systemic infections, kidney rejection, and death. The total number of complications for each patient was evaluated as well. In cases of complications, medical records were also investigated to exclude any other possible etiological factors. Between 2020 and 2021, patients who presented with fever were tested for Covid-19, and only patients with negative results were considered.

The inclusion criteria were: KT patients in the previous 5 years (2016–2021), a diagnosis of end-stage kidney disease (on dialysis and not on dialysis), agreement to be enrolled in the study and provision of a signed informed consent allowing the investigation of their medical and dental records for research purposes, completion of an oral examination with OPT within two months prior to KT, and the absence of other systemic pathology.

The exclusion criteria were: age under 18, inability to participate in the “dentistry supportive care” protocol after KT, refusal of consent to participate, inability to provide previous medical records and dental x-rays, and patients whose oral status changed between the pre- and post-KT periods. The sample was then divided into two additional groups based on the dental status prior to the surgery:

- Group Infected: patients with no dental clearance (patients had KT with the presence of at least one residual infectious foci in the oral cavity);
- Group Clear: patients with dental clearance (patients who had KT in the complete absence of infectious foci in the oral cavity).

These divisions were made upon evaluation of the OPT obtained prior to the transplantation.

### Microbiological analysis

For all patients who experienced systemic infections, medical records were retrospectively reviewed to check if microbiological analyses were performed by blood test and culture at the time when the complication occurred. If available, the bacteria detected during the blood test was reviewed to determine whether common oral bacteria were systemically present at the moment of the systemic infection.

### Statistical analysis

Group descriptive statistics are given as a number (percentage), for categorical variables, or as a mean ± SD (standard deviation), for continuous variables. The comparisons between the groups for the quantitative variables were made using the student’s t-test for two independent samples if the variables were normally distributed. Otherwise, the medians were compared using the Mann–Whitney U test. The comparison between the two groups for the qualitative variables was made by comparing the means using the chi-square test, or with Fisher’s exact test, in the case of at least one cell with an expected value < 5. All tests performed were two-sided, and the significance level was set at 5% (*p* = 0.05).

## Results

An initial total of 91 patients were examined in the oral surgery department at the C.I.R. Dental School, in Turin, Italy. There were 14 patients whose pre-transplantation dental and/or nephrological medical records were not found; they were consequently excluded. In total, 77 patients met the inclusion criteria: 47 females and 30 males, between the ages of 29 and 80 years (mean 53 ± 13 years). The characteristics of the sample are described in Table 1.

**Table 1:** Characteristics of the sample

Characteristic	Total (N = 77)	Infected (N = 19)	Clear (N = 58)	Infected versus clear, *P* value
Gender [Female]	34 (44%)	8 (42%)	26 (45%)	0.5
Age at KT [years]	53 ± 13 [29–80]	54 ± 13.5 [29–79]	53 ± 13.7 [33–80]	0.77
< 60 years	49 (64%)	14 (74%%)	35 (60%)	0.22
Followed at the C.I.R. Dental School before KT	35 (45%)	11 (58%)	20 (34%)	0.06
Age at study [years]	63 ± 13 [33–87]	62 ± 13 [40–84]	64 ± 13 [33–87]	0.56
Pathology				
Chronic kidney failure (stage III)	64 (83%)	13 (68%)	51 (88%)	0.06
Polycystic kidney	7 (9%)	4 (21%)	3 (5%)	0.22
Other	6 (8%)	2 (11%)	4 (7%)	0.46
Previous dialysis treatment	75 (97%)	1 (5%)	1 (2%)	0.43

Of the 77 patients who met the inclusion criteria, 35 were already followed at the C.I.R. Dental School before the KT, while the remaining 42 patients visited the oral surgery department only after KT. Group Infected was composed of 19 (25%) patients (those who had KT with the persistence of at least one residual infectious focus in the oral cavity), and Group Clear was composed of 58 (75%) patients (those who had KT in the complete absence of infectious foci in the oral cavity). Concerning the patients of Group Infected, the oral infectious foci untreated before transplantation were: 22 cavities, 5 cavities that required endodontic treatment, and 1 periodontal tooth that required extraction.

Of the 19 subjects who had untreated oral infectious foci present at the time of transplantation (Group Infected), 13 (68%) developed complications within 100 days of transplantation, and 11 (58%) did so after 100 days. Patients in Group Infected had a statistically significant increase in episodes of fever (*p* = 0.03), compared to those in Group Clear, with a higher relative risk (RR) of 3.66 in the first 100 days after transplantation.

Of the 58 subjects who arrived for KT without infectious foci (Group Clear), 31 (53%) had complications within 100 days of the transplant, and 23 (40%) did after 100 days.

For the group of patients who experienced complications, each patient experienced only one complication; no patients experienced multiple complications. The complications observed are summarized in Table 2.

**Table 2:** Systemic complications

Systemic complications	Infected (N = 19)	Clear (N = 58)	Infected versus clear, *P* value	Relative risk [95% CI]
Complications per patient (pt) within 100 days	0.9 ± 0.9 [0–3]	0.9 ± 1 [0–4]	0.99	
Complications per pt after 100 days	0.9 ± 1.4 [0–6]	0.9 ± 1.4 [0–6]	0.99	
Fever	9 (37%)	12 (21%)	0.27	2.4 [1.4–5.1]
Within 100 days	6 (26%)	5 (10%)	0.03	3.6 [0.9–4.7]
After 100 days	3 (16%)	7 (12%)	0.97	1.31[0.4–3.2]
Pneumonia	4 (21%)	1 (2%)	0.02	3.8 [2.1–7.2]
Within 100 days	2 (11%)	1 (2%)	0.15	2.9 [1.2–7.2]
After 100 days	2 (11%)	0 (0%)	0.06	4.4 [2.9–6.7]
Urinary tract infections	9 (47%)	34 (58%)	0.27	0.7 [0.3–1.5]
Within 100 days	6 (32%)	20 (34%)	0.52	0.9 [0.4–1.9]
After 100 days	3 (16%)	14 (24%)	0.34	0.6 [0.2–2]
Systemic infections	4 (21%)	11 (17%)	0.56	1.1 [0.4 – 2.8]
Within 100 days	3 (16%)	7(12%)	0.47	1.3 [0.4–4.6]
After 100 days	1 (5%)	4 (7%)	0.64	0.8 [0.09–6.4]
Rejection	1 (19%)	3 (5%)	0.69	1.0 [0.1–9.2]
Within 100 days	0	2 (3%)	0.57	
After 100 days	1 (5%)	1 (2%)	0.44	3.1 [0.2–46]

The table indicates the number and percentage of patients who experienced systemic complications after kidney transplantation. Group Infected includes patients who went to transplant with persistence of at least one residual infectious focus in the oral cavity. Group Clear includes patients who arrived for transplantation in the complete absence of infectious foci in the oral cavity

### Microbiological analyses

The main bacteria detected were *Klebsiella pneumoniae*, *Escherichia coli*, *Enterococcus faecalis*, *Staphylococcus epidermidis*, *Staphylococcus hominis*, *Staphylococcus warneri*, *Staphylococcus lugdunensis*, *Pseudomonas aeruginosa*, and *Proteus mirabilis*.

In detail, within 100 days of the transplant, 16% of Group Infected who had systemic infections presented with *Escherichia coli* or *Cytomegalovirus*, and 14% of Group Clear who had systemic infections presented with *Klebsiella pneumoniae, Cytomegalovirus, Escherichia coli, Polyomavirus, Morganella morganii,* or *Staphylococcus epidermidis* and the fungus *Candida albicans.*

More than 100 days after transplant, Group Infected had a single case (5%) of systemic infection, which was detected as *Cytomegalovirus*. In Group Clear, there were 5 patients (9%) who presented systemic infections, with *Staphylococcus epidermidis, Cytomegalovirus, Klebsiella pneumoniae*, and *Escherichia coli.*

No common oral bacteria [12, 24] were detected in any patients.

## Discussion

The first aim of this retrospective pilot study was to investigate a possible correlation between systemic complications and any missed oral infectious foci in patients who had undergone KT. The sample was divided into two groups, separating patients without dental clearance (with residual oral infectious foci prior to the transplantation) from patients with dental clearance, to assess whether latent oral infections may lead to systemic post-transplant complications. By examining the number of complications of patients in Group Infected (not cleared before transplant) and Group Clear (cleared before transplant), it was possible to calculate the relative risk for each category of complications (fever, pneumonia, urinary tract infections, systemic infections, rejection, death) within and after 100 days of the transplantation. A study period of 100 days was chosen as the cut off between early and late complications according to what has been described in the literature [25]. No patient in either group died, but most of the patients examined had urinary tract infections (about 50% of the total had complications).

From the analysis of the various relative risks derived from the relationship between Group Infected and Group Clear for each complication studied, a statistically significant difference was found concerning the onset of fever within 100 days after KT (RR = 3.66, *p* = 0.03). Since the RR > 1, the risk of occurrence of the event (fever within 100 days) in Group Infected was higher than in Group Clear; however, no statistical results were identified concerning any of the other complications. When considering complications in total (without the division between early and late complications), a significant statistical difference was also found for pneumonia (*p* = 0.02). However, patients who experienced pneumonia were very limited (4 patients in Group Infected vs. 1 patient of Group Clear), making the statistical analysis unreliable. Since a statistical difference was found between Group Infected and Group Clear, the first null hypothesis was rejected. However, the results from the present study should be read with caution.

The main limitation of this study is related to its small sample size. It is important to note that different results may be produced when considering a larger sample size. Other limitations of the study are that the data were not correlated to the age of the patient, and the time of the study was limited. Nonetheless, no data about the oral hygiene of the patients, which may be associated with poor education, self-care, and lower socio-economic status, were recorded and no detailed data about pre- and post-transplant antimicrobial prophylaxis were retrospectively found. No other associations were noticed between the infected group and urinary tract infections, rejection, or systemic infections. However, considering all of the above-mentioned limitations, the results of the 5-year pilot study, together with the debate on the topic that remains open in the literature, pave the way to continue the study by improving its sample size and conducting further research. Indeed, the present results should be confirmed in prospective studies.

In a patient with chronic kidney failure, KT is certainly the gold standard treatment, since it allows the achievement of a higher survival rate, better quality of life, and a lower consumption of economic and health resources [3, 26]. Despite significant improvements in immunosuppressive therapies, surgical techniques, and medical care, transplant patients still face a moderate risk for infection, septicemia, and rejection in the post-transplant period [27, 28].

Considering the oral cavity as a possible site of infectious foci, it is possible that the latter may have a role in the post-KT course of treatment, but currently there are no longitudinal studies of the short post-transplant period to evaluate the impact of oral health. Indeed, there is controversial scientific evidence regarding the role of chronic odontogenic infections and inflammations and how they can increase the risk of hospitalization in KT patients.

Zwiech and Bruzda-Zwiech [21] analyzed 91 post-KT patients over five years. Data from their study showed that poor oral clearance is associated with an increased risk of systemic complications during the first year after transplantation. Moest et al. [29] concluded that dental treatments are fundamental for improving the post-surgical course after transplantation. Blach et al. [30] found an association between chronic periodontitis and the risk of death after KT, based on an observational period of five years. In agreement, consistencies were shown by Nunes-Dos-Santos et al. [31], who found in a recent systematic review a correlation between periodontal disease and decreased kidney graft functionality. Nonetheless, in 2022 Gomes et al. [32] suggested a possible role of periodontitis in the onset of hyperglycemia in KT patients. By contrast, results from different studies are in disagreement with the above-mentioned conclusions. Data from a study by Schander et al. [33] did not support the correlation between oral health infections and post-KT failure. Wynimko et al. [34], in disagreement with Blach et al. [30] and Nunes-Dos-Santos et al. [31], failed to demonstrate that chronic periodontitis may be associated with an increased risk of complications in KT patients.

Research is continuing to focus on the effect that oral health status may have on the general systemic health of patients [35–38]. However, a final demonstration of an effective correlation between oral health status and complications in KT patients remains a matter of discussion, and more studies on the topic are needed.

Additionally, the second aim of the present study was to investigate whether or not typical pathogenic bacteria of the oral cavity were present in transplant patients who experienced systemic infections. From the data obtained in the present study, no bacteria that typically swarm the oral cavity were detected in any of patients analyzed. Consequently, the second null hypothesis was accepted. However, since the type of bacteria depends on complex interactions between commensal and/or opportunist bacteria [39, 40], it may be interesting to compare bacteria that are systemically present before the KT with those that are present if a systemic infection occurs after KT. In fact, some of the detected bacteria and fungi, such as *Candina*, *Morganella morganii*, *Staphylococcus hominis*, and *Staphylococcus epidermidis*, can be present as opportunist in the oral cavity even in patients without chronic kidney disease [41]. Comparing bacteria that are systemically present before KT with those present after may be possible by performing an antibiogram both prior and after surgery in patients with and without dental clearance in a prospective study. This could allow further investigation into whether the presence of untreated oral infectious foci can statistically influence the bacteria population in the event that a systemic infection occurs after KT.

In conclusion, aside from all the limitations of this study, the results have shown a statistically significant association between the presence of residual oral foci pre-KT and the onset of fever within 100 days of the transplant itself. This may confirm that systemic complications could be linked to infections originating from the oral cavity. Even if only one statistically significant datum has been identified, it is possible to speculate that it would be better to undergo KT in the absence of oral infectious foci. In fact, post-transplant infections can generally lead to more or less serious complications, which in the worst cases can lead to the death of the patient. For this reason, it is essential to ensure that there is an absence of infectious processes that could be nested throughout the body prior to undergoing a transplant. It can be hypothesized that, even in the absence of a direct correlation between infectious foci of the oral cavity and complications brought on by oral pathogens, a pro-inflammatory situation still remains that may determine an increased susceptibility of the immunosuppressed patient to the onset of other infections at the systemic level.

## Conclusion

Within the limitations of this retrospective pilot study, and based on the results, a correlation between the absence of dental clearance prior to KT and a higher RR of developing fever within the first 100 days post transplantation was highlighted. The results of the study encourage the continuation of research on the topic, which remains controversial. Further prospective research is needed to determine whether dental clearance should be obtained prior to KT operations to reduce the risk of post-transplant infections that might originate from the oral cavity sources.

## Data Availability

All data and materials are available from the corresponding author upon reasonable request.

## Abbreviations

KT: Kidney transplant
OPT: Orthopantomography
RR: Relative risk

## References

[1] Wang AY, Bellomo R (2018). Renal replacement therapy in the ICU: intermittent hemodialysis, sustained low-efficiency dialysis or continuous renal replacement therapy?. Curr Opin Crit Care.

[2] Hwang SD, Lee JH, Lee SW, Park KM, Kim JK, Kim MJ, Song JH (2018). Efficacy and safety of induction therapy in kidney transplantation: a network meta-analysis. Transplant Proc.

[3] Liyanage T, Ninomiya T, Jha V, Neal B, Patrice HM, Okpechi I, Zhao MH, Lv J, Garg AX, Knight J, Rodgers A, Gallagher M, Kotwal S, Cass A, Perkovic V (2015). Worldwide access to treatment for end-stage kidney disease: a systematic review. Lancet.

[4] Lim MA, Kohli J, Bloom RD (2017). Immunosuppression for kidney transplantation: where are we now and where are we going?. Transplant Rev.

[5] Radochová V, Šembera M, Slezák R, Heneberk O, Radocha J (2021). Oral mucositis association with periodontal status: a retrospective analysis of 496 patients undergoing hematopoietic stem cell transplantation. J Clin Med.

[6] Memikoğlu KO, Keven K, Sengül S, Soypaçaci Z, Ertürk S, Erbay B (2007). Urinary tract infections following renal transplantation: a single-center experience. Transplant Proc.

[7] Angelico R, Pellicciaro M, Venza F, Manzia TM, Cacciola R, Anselmo A, Toti L, Monaco A, Iaria G, Tisone G (2021). Urological complications in kidney transplant recipients: analysis of the risk factors and impact on transplant outcomes in the era of “extended criteria donors”. Transplantology.

[8] Kawecki D, Wszola M, Kwiatkowski A, Sawicka-Grzelak A, Durlik M, Paczek L, Mlynarczyk G, Chmura A (2014). Bacterial and fungal infections in the early post-transplant period after kidney transplantation: etiological agents and their susceptibility. Transplant Proc.

[9] Sabagh M, Mohammadi S, Ramouz A, Khajeh E, Ghamarnejad O, Morath C, Mieth M, Kulu Y, Zeier M, Mehrabi A, Golriz M (2021). Validating consensus-defined severity grading of lymphatic complications after kidney transplant. J Clin Med.

[10] Oliveira SG, Nishiyama RR, Trigo CAC, Mattos-Guaraldi AL, Dávila AMR, Jardim R, Aguiar FHB (2021). Core of the saliva microbiome: an analysis of the MG-RAST data. BMC Oral Health.

[11] Hall MW, Singh N, Ng KF, Lam DK, Goldberg MB, Tenenbaum HC, Neufeld JD, Beiko RG, Senadheera DB (2017). Inter-personal diversity and temporal dynamics of dental, tongue, and salivary microbiota in the healthy oral cavity. NPJ Biofilms Microbiomes.

[12] Handsley-Davis M, Jamieson L, Kapellas K, Hedges J, Weyrich LS (2020). The role of the oral microbiota in chronic non-communicable disease and its relevance to the Indigenous health gap in Australia. BMC Oral Health.

[13] Jia G, Zhi A, Lai PFH, Wang G, Xia Y, Xiong Z, Zhang H, Che N, Ai L (2018). The oral microbiota: a mechanistic role for systemic diseases. Br Dent J.

[14] Dominy SS, Lynch C, Ermini F, Benedyk M, Marczyk A, Konradi A, Nguyen M, Haditsch U, Raha D, Griffin C (2019). *Porphyromonas gingivalis* in Alzheimer’s disease brains: evidence for disease causation and treatment with small-molecule inhibitors. Sci Adv.

[15] Bergandi L, Giuggia B, Alovisi M, Comba A, Silvagno F, Maule M, Aldieri E, Scotti N, Scacciatella P, Conrotto F, Berutti E, Pasqualini D (2019). Endothelial dysfunction marker variation in young adults with chronic apical periodontitis before and after endodontic treatment. J Endod.

[16] Fiorillo L, De Stefano R, Cervino G, Crimi S, Bianchi A, Campagna P, Herford AS, Laino L, Cicciù M (2019). Oral and psychological alterations in haemophiliac patients. Biomedicines.

[17] Cervino G, Fiorillo L, Laino L, Herford AS, Lauritano F, Giudice GL, Famà F, Santoro R, Troiano G, Iannello G, Cicciù M (2018). Oral health impact profile in celiac patients: analysis of recent findings in a literature review. Gastroenterol Res Pract.

[18] Wilson RL, Martinez-Tirado J, Whelchel J, Lordon RE (1982). Occult dental infection causing fever in renal transplant patients. Am J Kidney Dis.

[19] Schmalz G, Patschan S, Patschan D, Ziebolz D (2020). Oral health-related quality of life in adult patients with end-stage kidney diseases undergoing renal replacement therapy: a systematic review. BMC Nephrol.

[20] Sarmento DJS, Caliento R, Maciel RF, Braz-Silva PH, Pestana JOMA, Lockhart PB, Gallottini M (2020). Poor oral health status and short-term outcome of kidney transplantation. Spec Care Dentist.

[21] Zwiech R, Bruzda-Zwiech A (2013). Does oral health contribute to post-transplant complications in kidney allograft recipients?. Acta Odontol Scand.

[22] Patton LL (2019). The fallacy of pre-kidney transplantation "dental clearance". Oral Surg Oral Med Oral Pathol Oral Radiol.

[23] Schönfeld B, Varga Á, Szakály P, Bán Á (2019). Oral health status of kidney transplant patients. Transplant Proc.

[24] Mosaddad SA, Tahmasebi E, Yazdanian A, Rezvani MB, Seifalian A, Yazdanian M, Tebyanian H (2019). Oral microbial biofilms: an update. Eur J Clin Microbiol Infect Dis.

[25] Kazımoğlu H, Harman R, Mercimek MN, Dokur M, Uysal E (2018). Evaluation of early and late-term infections after renal transplantation: clinical experiences of Sanko University Medical Faculty Transplantation Center. Turk J Urol.

[26] Zhang Y, Zhang F, Zhang W, Pan J, Wu X, Liao G, Zhang X (2021). Kidney transplantation improve semen quality in patients with dialysis: a systematic review and meta-analysis. Andrologia.

[27] Wan SS, Ying TD, Wyburn K, Roberts DM, Wyld M, Chadban SJ (2018). The treatment of antibody-mediated rejection in kidney transplantation: an updated systematic review and meta-analysis. Transplantation.

[28] Jones-Hughes T, Snowsill T, Haasova M, Coelho H, Crathorne L, Cooper C, Mujica-Mota R, Peters J, Varley-Campbell J, Huxley N, Moore J, Allwood M, Lowe J, Hyde C, Hoyle M, Bond M, Anderson R (2016). Immunosuppressive therapy for kidney transplantation in adults: a systematic review and economic model. Health Technol Assess.

[29] Moest T, Lutz R, Jahn AE, Heller K, Schiffer M, Adler W, Deschner J, Weber M, Kesting MR (2022). Frequency of the necessity of dentoalveolar surgery or conservative treatment in patients before kidney transplantation depending on the duration of dialysis and causative nephrological disease. Clin Oral Investig.

[30] Blach A, Franek E, Witula A, Kolonko A, Chudek J, Drugacz J, Wiecek A (2009). The influence of chronic periodontitis on serum TNF-alpha, IL-6 and hs-CRP concentrations, and function of graft and survival of kidney transplant recipients. Clin Transplant.

[31] Nunes-Dos-Santos DL, Gomes SV, Rodrigues VP, Pereira ALA (2020). Periodontal status and clinical outcomes in kidney transplant recipients: a systematic review. Oral Dis.

[32] Gomes SV, Rodrigues V, Nunes-Dos-Santos DL, Pereira ALA, Peres MA (2022). The relationship between periodontal status and hyperglycemia after kidney transplantation. Clin Oral Investig.

[33] Schander K, Jontell M, Johansson P, Nordén G, Hakeberg M, Bratel J (2009). Oral infections and their influence on medical rehabilitation in kidney transplant patients. Swed Dent J.

[34] Wynimko M, Walicka M, Sanchak Y, Gozdowski D, Błach A, Więcek A, Śliwczyński A, Franek E, Kolonko A (2020). Influence of chronic periodontitis on the long-term mortality and cardiovascular events in kidney transplant recipients. J Clin Med.

[35] Mekhdieva E, Del Fabbro M, Alovisi M, Comba A, Scotti N, Tumedei M, Carossa M, Berutti E, Pasqualini D (2021). Postoperative pain following root canal filling with bioceramic vs. traditional filling techniques: a systematic review and meta-analysis of randomized controlled trials. J Clin Med.

[36] Pasqualini D, Scotti N, Ambrogio P, Alovisi M, Berutti E (2012). Atypical facial pain related to apical fenestration and overfilling. Int Endod J.

[37] Schmalz G, Garbade J, Sommerwerck U, Kollmar O, Ziebolz D (2021). Oral health-related quality of life of patients after solid organ transplantation is not affected by oral conditions: results of a multicentre cross-sectional study. Med Oral Patol Oral Cir Bucal.

[38] Schmalz G, Ziebolz D (2020). Special issue “oral health and systemic diseases”. J Clin Med.

[39] Paharik AE, Horswill AR (2016). The staphylococcal biofilm: adhesins, regulation, and host response. Microbiol Spectr.

[40] Kiedrowski MR, Horswill AR (2011). New approaches for treating staphylococcal biofilm infections. Ann N Y Acad Sci.

[41] Ohara-Nemoto Y, Haraga H, Kimura S, Nemoto TK (2008). Occurrence of staphylococci in the oral cavities of healthy adults and nasal oral trafficking of the bacteria. J Med Microbiol.

